# Evaluating critical success factors in implementing E-learning system using multi-criteria decision-making

**DOI:** 10.1371/journal.pone.0231465

**Published:** 2020-05-04

**Authors:** Quadri Noorulhasan Naveed, Mohamed Rafik Noor Qureshi, Nasser Tairan, AbdulHafeez Mohammad, Asadullah Shaikh, Alhuseen O. Alsayed, Asadullah Shah, Fahad Mazaed Alotaibi

**Affiliations:** 1 College of Computer Science, King Khalid University, Abha, Saudi Arabia; 2 College of Engineering, King Khalid University, Abha, Saudi Arabia; 3 Department of Computer Science, Bahria University, Lahore, Pakistan; 4 College of Computer Science and Information Systems, Najran University, Najran, Saudi Arabia; 5 Deanship of Scientific Research, King Abdulaziz University, Jeddah, Saudi Arabia; 6 Kulliyyah of ICT, International Islamic University Malaysia, Kuala Lumpur, Malaysia; 7 Department of Information Technology, Faculty of Computing and Information Technology, King Abdulaziz University, Jeddah, Saudi Arabia; Shandong University of Science and Technology, CHINA

## Abstract

Learning using the Internet or training through E-Learning is growing rapidly and is increasingly favored over the traditional methods of learning and teaching. This radical shift is directly linked to the revolution in digital computer technology. The revolution propelled by innovation in computer technology has widened the scope of E-Learning and teaching, whereby the process of exchanging information has been made simple, transparent, and effective. The E-Learning system depends on different success factors from diverse points of view such as system, support from the institution, instructor, and student. Thus, the effect of critical success factors (CSFs) on the E-Learning system must be critically analyzed to make it more effective and successful. This current paper employed the analytic hierarchy process (AHP) with group decision-making (GDM) and Fuzzy AHP (FAHP) to study the diversified factors from different dimensions of the web-based E-Learning system. The present paper quantified the CSFs along with its dimensions. Five different dimensions and 25 factors associated with the web-based E-Learning system were revealed through the literature review and were analyzed further. Furthermore, the influence of each factor was derived successfully. Knowing the impact of each E-Learning factor will help stakeholders to construct education policies, manage the E-Learning system, perform asset management, and keep pace with global changes in knowledge acquisition and management.

## 1. Introduction

E-Learning is a state-of-the-art methodology for learning and teaching in digital environments aimed at improving education through enhancing the teaching and learning processes [1, 2]. E-Learning systems, which are exempt from time and location limitations, offer opportunities for teaching and learning and play a vital role in promoting new teaching methods [3]. It is observed that various developed and developing countries have adopted and implemented E-Learning for Teaching-Learning. It may include complete dependency through the learning management system (LMS), blended E-Learning or Traditional blackboard Teaching-Learning with E-Learning.

E-Learning is recognized as a key factor in enhancing the performance of the education sector and its stakeholders’ satisfaction. Research has shown that E-Learning provides improved ability to share information, cost-efficacy, accessibility, and easy access through the World Wide Web (www). Not only that, E-Learning supports every student in a new learning approach to achieve a high volume of interaction and an easy learning environment with the help of technology integration in education [4, 5]. Several studies have also discussed E-Learning as a common methodology that is becoming user-friendly as it provides relaxed access and usage [6, 7]. Furthermore, E-Learning allows students to study and learn freely using cutting-edge new technology without conventional teaching approaches like direct supervision and control mechanisms [8–10]. The fast development in IT with new software and high configuration of hardware have made E-Learning more easy and useful, which then improves university learning outcomes [11]. It is therefore indisputable that E-Learning has become an indispensable tool in educational technology [12].

Despite its advantages, E-Learning’s full and successful implementation is yet to be achieved [13]. If E-Learning is successfully implemented into the education system, its many perceived benefits can be noticed. Past studies have indicated that critical success factors (CSFs) play a vital role in the successful implementation of E-Learning. Furthermore, it has also been revealed that the critical factors of different dimensions can have varying effects on the E-Learning system [14–16]. Therefore, it is imperative to explicitly investigate the assessment and prioritisation of E-Learning’s CSFs and propose a hierarchical model of E-Learning’s success factors. Such a model will allow E-Learning administrators and other stakeholders to recognise and start paying attention to the most important E-Learning success factors. The E-Learning needs a huge investment by the management to implement in the education system. It has been studied that despite huge investment the E-Learning system usage is still low [17]. Many researchers have attempted to resolve various issues like the impact of (CSFs) on the E-Learning system, issues concerning user satisfaction, and the effect of the E-Learning system on student learning [3]. To implemnet the E-Learning system effectively, it is significant to know the CSFs that plays the vital role in successful implementaion fo E-Learning. There is little work on prioritisation and ranking of the CSFs.

To address the discussed research gaps, this current study attempts to evaluate and rank the dimensions and CSFs of the E-Learning system. An analytic hierarchy process with group decision making (AHP-GDM) and Fuzzy AHP (FAHP) based multi-criteria decision-making (MCDM) is used in the assessment and prioritization of dimensions and CSFs of the E-Learning system. The present research is undertaken to accomplish three major objectives, namely:

1. To conduct a comprehensive survey of the E-Learning dimensions and CSFs through an in-depth literature review.
2. To prioritize the E-Learning dimensions and CSFs using MCDM in crisp and fuzzy environment.
3. To provide useful recommendations based on the prioritized dimensions and CSFs of E-Learning.

This paper is organized as follows. Section 2 presents the review of literature on CSF of E-Learning and MCDM based methodology in E-Learning. Section 3 discuss the framework to identify the CSFs of E-Learning, Next, Section 4 provides the AHP-GDM and FAHP research methodologies, while the application of AHP-GDM and FAHP for prioritizing E-Learning’s CSFs are presented in Section 5. Section 3 discusses the results of the prioritization, whereas Section 7 discusses the limitations of the present study and provides scope for the future research, Section 8 concludes the present study. Finally, a detailed Recommendation of Effective E-Learning Implementation for Students, Instructors and Developers is provided in the last Section 9.

## 2. Literature review

CSFs are commonly used for the seamless execution of various strategies and programs by business establishments. It refers to ‘the limited number of objectives areas will ensure the organization’s successful competitive performance if they are satisfactory’ [18]’ and demands the continuous attention of managers in the selected areas. CSFs encompasses the crucial factors that are critical in the areas where performance is very important to establish the success of organizations [19]. As such, there should be a strong focus on these factors to achieve success [20]. Despite the crucial importance of dimensions and CSFs for organizational success, the limited research that may help in the successful implementation of E-Learning has been undertaken [21].

Several CSFs influence effective teaching and learning methodologies in E-Learning. Many dimensions and CSFs of E-Learning have a significant effect on the teaching-learning process. Prior studies have reported on important E-Learning CSFs. For example, a study [3] focused on effective E-Learning factors in Saudi universities through a systematic review and statistical analysis of academic staff and students’ perspectives. The study concluded that CSFs are the most effective factors for the successful implementation of E-Learning. Bhuasiri [17] studied and analyzed the CSF for E-Learning in developing countries. Abdullah and Rowley [3] carried out comparative perspective study on E-Learning CSF. Hence, CSFs must be thoroughly studied. The analysis of the dimensions and CSFs will guarantee the effective implementation of the E-Learning system.

Apart from identifying the various significant CSFs of E-Learning, a detailed literature review on various MCDM-based modeling has also been carried out and is presented in this section. Several scholars have utilized MCDM to examine the E-Learning system. For instance, FAHP was applied in a study [22] to propose a framework for learning with the help of Massive Open Online Courses (MOOC). This framework can help in increasing the effectiveness of teaching. Moreover, learners can get simplified lifelong learning with maximized motivation, while simultaneously the number of dropouts can be reduced. Other than that, a recent study [23] established a model for evaluating the contents of multimedia quality in E-Learning by calculating each attribute’s priority weights using the MCDM-based Analytic Network Process (ANP).

On the other hand, FAHP has also been used in investigating the possibility of using a new PVM-VSI method considering the variants in different criteria [24]. Another study [25] used the AHP-GDM methodology to derive a strategic plan for evaluating E-Learning applications using five different approaches at different levels. The researcher proposed a quantitative evaluation method for all the E-Learning decision-makers (DMs) from higher education institutes. Next, FAHP was employed to rank various E-Learning factors to achieve E-Learning performance. The five most important factors from the students as well as the instructors’ point of view were derived [26]. Research on E-learning website selection and ranking have been carried using fuzzy Complex Proportional Assessment (COPRAS) [27], weighted distance-based approximation [28], multi attribute decision-making approaches [29], multi-attribute decision-making matrix methodology [30] and FAHP, COPRAS, VlseKriterijumska Optimizacija I Kompromisno Resenje (VIKOR), Weighted Distance Based Approximation (WDBA) [31].

The in-depth review of the literature also revealed that many researchers have combined different methodologies to get more accurate results. For example, FAHP and Step-wise Weight Assessment Ratio Analysis (SWARA) methods were applied to assess five dimensions and 24 critical E-Learning factors [32]. On the other hand, another study used AHP in the selection of software-based learning objectives (LO). SDUNESA, a web-based software, utilized the AHP method parameter for the LO selection. The implemented methodology reduced the time for searching LO in large database systems [33]. Apart from that, the research carried out by [17] applied a combined approach of MCDM to prioritize and identify E-Learning CSFs. This research found the most successful factors based on their importance level using the feedback from several stakeholders such as ICT experts and staff members. In another study [34] investigated the online learning quality using the FAHP method to study Internet resources and their environment. This study analyzed Internet learning quality using qualitative and quantitative methodologies.

Next, Kang et al. [35] used AHP to examine the different characteristics of the E-Learning system and applied the Technique for Order of Preference by Similarity to Ideal Solution (TOPSIS) in ranking different criteria using derived weights. The researchers stressed that the integration of TOPSIS with AHP can be beneficial in real usage. In their study, 33 criteria for successful implementation were revealed and the AHP methodology was utilized in creating a hierarchical model and assessment of adopted E-Learning CSFs.

Based on the reviewed literature, the combination of several methods have been revealed to be much more effective and various researchers have applied it for analyzing different E-Learning features. Therefore, this current study’s focus on AHP-GDM and FAHP. The employed methods will contribute to the existing literature on to E-Learning.

## 3. Framework for the identification of CSFs in E-Learning

Numerous studies have established key findings concerning E-Learning. Among them, many have critically examined the dimensions and CSFs of the E-Learning system. Some studies [5], [14, 36] have classified the CSFs of E-Learning into different dimensions such as Institutional Management Service, Instructors, System and Technological, Students, and Content Design. In the first stage of this study, all possible success factors related to E-Learning were identified which resulted in 36 CSFs. Next, after critically reviewing the identified CSFs with the help of DMs, these factors were reduced to 25 and then grouped into five dimensions. The identified CSFs are further explained in this section.

The following steps were employed to find and categorize the important CSFs that affect the E-Learning system:

- A thorough literature review to collect the dimensions and CSFs of E-Learning.
- Identification of CSFs commonly used in E-Learning research.
- Utilisation of AHP-GDM and FAHP methodologies to assess, evaluate, and prioritise E-Learning CSFs.

**Fig 1:**
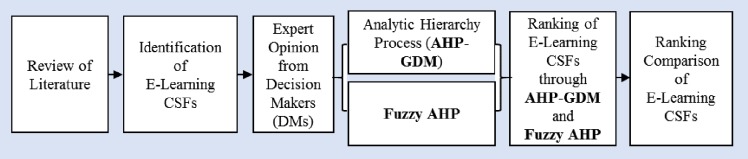
AHP-GDM and FAHP based framework for ranking CSFs of E-Learning.

Based on the above steps, the selection framework of CSFs is illustrated in Fig 1. The AHP-GDM and FAHP methodologies were employed to prioritize the selected CSFs. Table 1 lists the chosen factors and their dimensions.

**Table 1:** Dimensions and CSFs of E-Learning.

Dimensions	CSFs	References
Students’ Dimension	Attitude towards E-Learning (ATE)	[5, 39, 41, 40, 45, 48–52]
	Students’ Motivation (SM)	[3, 48, 53–57]
	General Internet self-efficacy (GIS)	[5, 39, 41, 47, 48, 49, 51, 52, 53, 55, 58, 59, 60, 61]
	Interaction With Other Students (IOS)	[39, 40, 51, 58, 59, 62–64]
	Commitment towards Online Studies (CTO)	[38, 40, 54, 55, 65]
Instructors’ Dimension	Instructors’ Attitude towards E-Learning (IAT)	[5, 45, 48, 50, 54, 55, 64, 66–68]
	Instructors’ ICT skills (IIS)	[3, 11, 39, 40, 48, 49, 51, 53–55, 57–67]
	Easy Language Communication (ELC)	[40, 50, 56, 69]
	Appropriate timely Feedback	[5, 40,41, 45, 49, 54, 70]
Design and Contents’ Dimension	Interactive Learning Activity (ILA)	[39, 40, 42, 49, 63, 71]
	Appropriate Course Design (ACD)	[39, 40, 50, 72]
	Use of Multimedia Instruction (UMI)	[67, 70, 71]
	User–Friendly Organized (UFO)	[40, 41–43, 54, 73]
	Understandable Content	[40, 43, 44, 49, 54, 55, 61]
System and Technological Dimension	Appropriate System (AS)	[44, 50, 52, 61, 70, 74]
	Ease of Access (EoA)	[5, 11, 52, 72]
	Technical Support for Users (TSU)	[42, 45, 49, 51, 56, 70, 75, 76]
	Good Internet Speed (GIS)	[3, 41, 45, 48, 49, 52, 58, 60, 65, 70, 72]
	Efficient Technology Infrastructure (ETI)	[41, 46, 47, 50, 55, 58–60, 62, 69, 72, 75, 77–79]
	Reliability (R)	[3, 5, 40, 42, 43, 49, 50, 54, 55, 58, 65, 70, 75, 79]
Institutional Management Dimension	Infrastructure Readiness (IR)	[47, 50, 51, 54, 70, 80, 81]
	Financial Readiness (FR)	[40, 47, 54, 55, 75, 81, 82]
	Training of Users (SST)	[3, 50, 51, 75, 76, 82]
	Support for Faculty (SF)	[38, 48, 51, 53]
	Ethical & Legal Issues (ELI)	[5, 40]

### 3.1. Students’ dimension

Students are considered as one of the main stakeholders of the E-Learning system. It has been observed that students are given preference over the other stakeholders as they are the main beneficiary of the E-Learning system [5, 37, 38]. Moreover, students are expected to acquire assistance from the system and the system would be more successful and valuable if students use it appropriately. Meanwhile, students’ demand for diversified education has been growing. For instance, request for such education from non-traditional female students has increased considerably. E-Learning can play a crucial role in fulfilling such demands. Not only, but full-time working professionals may also take advantage of the E-Learning system as per their convenience. Thus, students’ dimension greatly influences the E-Learning system. The various CSFs from this dimension that influences the E-Learning system are listed in Table 1.

### 3.2. Instructors’ dimension

The instructors’ dimension concerns the satisfaction and gratification of students regarding their E-Learning experience. Instructors’ attitude and approach are critical in the efficient and productive implementation of the system. Moreover, their teaching style also plays a key role and provides a stimulating effect on the student’s recognition of E-Learning education [38]. Students are sure to engage in E-Learning if they find the instructors’ lectures useful and friendly with quality content. Additionally, instructors’ characteristics significantly decide the impact, effectiveness, and quality of educational LMS [39, 40]. Another study has highlighted that the result and conclusion of learning management systems are exaggerated by the teacher’s features, like opinion concerning the use of novel technology, teaching-learning styles, and mechanism of technology employed [41]. Several CSFs under the instructors’ dimensions are presented in Table 1.

### 3.3. Design and contents’ dimension

The dimension of content design has a direct effect on the success of E-Learning. Well-structured course contents, tools for learning, and activities and curriculum enable positive learning experiences. If a virtual course’s interface is user-friendly and the contents are clear, the learners’ interest and gratification will increase. Simple and properly arranged interfaces of virtual courses encourage students to take up those courses and learn via the Internet, according to their time, place, and flexibility. Many researchers [42, 43] have listed various important content design CSFs, which are mentioned in Table 1.

### 3.4. System and technological dimension

This dimension assumes a key part in conveying instructive teaching and learning through web technology [44] using different devices like video conferencing, sound, and content-based systems. System quality relates to the E-Learning website’s quality through which students can easily access learning materials of different courses [44]. The innovative system design with changing technological dimension helps in the rapid utilization of the devices [45]. For example, the broadcast speed of web-information impacts the students’ fulfillment, whereby the stacking pace of web-information remains specifically associated with the host server. The stacking pace of the online edge will be high if a preeminent class server is used [46]. Table 1 tabulates the various CSFs under the system and technological dimension.

### 3.5. Institutional management service dimension

The institutional management service dimension concerns the organizational support feature that makes E-Learning effective. Institutional support is critical in measuring the perceived satisfaction of all stakeholders [38, 47]. Infrastructure readiness, financial readiness, training of users, support for faculty, and ethical and legal issues are important CSFs of this dimension. The CSFs related to the institutional management service dimension are presented in Table 1.

## 4. Overview of MCDM based research methodologies

The present paper applies two research methodologies i.e. AHP-GDM and FAHP. The AHP has been widely used in solving problems containing multi-level hierarchical structures of objectives, criteria, sub-criteria, and alternatives. Since the decision making in AHP is carried out through the human judgment of DM, there lies a chance of bias decision making. The bias decision making may be restricted by using GDM hence a combinatorial approach of AHP-GDM will be fruitful. Further, the decision making may have vagueness, this may be removed by using fuzzy AHP. AHP-GDM and FAHP provide ease in pairwise comparison and fine-tuning the results, the detail steps are further described as follows:

### 4.1. Analytic hierarchy process (AHP)

In 1980, T.L. Saaty developed a systematic decision-support procedure known as AHP. The developed AHP process is capable of resolving the complex problems which may consist of multiple-criteria, multiple-levels, complex structure, etc. using a pairwise judgment from DMs. The use of AHP has been found in various researches in a wide variety of research problems [83–85].

The AHP uses the judgment of a DM in a pairwise comparison using Saaty’s nine-point scale as given in Table 2. The DM’s vast experience and deep knowledge related to the decision problem helps in a pairwise comparison. The single opinion from DM may be biased and misleading. Such a decision may not fulfill the requirement of the decision problem and thus becomes unusable in decision-making. The AHP may further be strengthened by using group decision-making (GDM). The use of GDM will improve the accuracy of decision-making. The GDM uses more DMs for resolving the given problem, thus it helps in delivering meaningful, robust and comprehensive single decision to solve the given decision problem.

**Table 2:** Saaty’s nine-point scale [86].

Intensity of Relative Importance	Definition
1	Equally preferred
3	Moderately preferred
5	Essentially preferred
7	Very strongly preferred
9	Extremely preferred
2,4,6,8	Intermediate importance between two adjacent judgement

The detailed AHP-GDM is discussed as follows:

***Step 1*:**

The various CSFs of E-Learning forming a single hierarchy are converted to a matrix form to construct a comparison matrix ‘*Q*’. The comparison matrix may also be termed as a decision matrix. In a pairwise comparison, the scale given in Table 2 is be applied to compare a pair of two CSFs presents in the ‘*Q*’ matrix. Thus, each entry in *Q* matrix is compared with respective entry to its level of importance. In other words, the element, *d*_*mn*_ of the ‘*Q*’ matrix, compares the *m*^*th*^ element with that of the *n*^*th*^ in terms of its importance level.

$$Q=\left[\begin{array}{cccc} d_{11} & d_{12} & \ldots & d_{1n}\\ d_{21} & d_{22} & \ldots & d_{2n}\\ \vdots & \vdots & \vdots & \vdots \\ d_{m1} & d_{m2} & \ldots & d_{mn} \end{array}\right]$$ (1)

***Step 2*:**

The ‘*Q*’ decision matrix is be arrived using a pairwise judgment from each DM participating in the group decision-making process. The overall geometric means (GM) of each pairwise decision is calculated. Thus the pairwise decision and subsequent priority vector (*PV*) are be calculated.

***Step 3*:**

In the process of AHP-GDM, the ‘*Q*’ decision matrix with the derived pairwise comparison entries, an overall summation of the product of the sum of each vector column for both the matrices with the *PV* values of each row is calculated. Later on, the principal eigenvalue (*λ*_*max*_), is be calculated as:

$$\lambda_{max}=\sum_{i,j=1}^{k}C_{j}{PV}_{i}$$ (2)

where *c*_*j*_ is the sum of each column vector.

***Step 4*:**

In the AHP-GDM, the acceptance of decision is based on the decision consistency of DMs. Hence it becomes mandatory to verify the decision consistency in the decision problem. From the derived ‘*Q*’ decision matrix, the consistency index (CI) is calculated by using Eq (3):

$$CI=\frac{\lambda_{max}-n}{n-1}$$ (3)

Where CI = consistency index and n = the number of elements of each of the matrix.

***Step 5*:**

The consistency ratio is calculated using the random index. The random index (RI) can be calculated using Eq (4).

$$RI=\frac{{1.98(n-2)}^{}}{n}$$ (4)

***Step 6*:**

The value of consistency ratio (CR) is significant to decide whether the derived a pairwise judgmental matrix is acceptable or not. Based on the CR value the matrix is accepted if the obtained CR value is less than 10%. In the case of the obtained value is more than 10%, the pairwise judgmental decision is revised. The CR may be obtained using Eq (4).

$$CR=\frac{CI}{RI}$$ (5)

***Step 7*:**

In a pairwise comparison of decision matrix “*Q*”, the fuzzy scale based on TFN is used, Which is shown in Table 10. The fuzzy scale may help in getting the pairwise comparison matrices (*A*_*i*_,*i* = 1,2,…,*n*). Based on the importance level, DMs may fix the relevant relationship and suitable choose the scale to assign a weight to these matrices.

### 4.2. Fuzzy Analytic Hierarchy Process (FAHP)

In the FAHP, a fuzzy set theory along with extension principle can be applied. The use of FAHP is considered indispensable when the inaccuracy in decision-making has to be removed. Many a time in decision-making, manual judgment is critical. However, while making a human judgment there is a possibility of making an error in judgmental decisions. The DM may become bias in his decision making. To reduce such error often the fuzzy methodology is applied in decision-making [87]. The basic fuzzy set theory and extension principles help in accurate decision making. The basic fuzzy set theory and extension principles are further described.

#### 4.2.1. Fuzzy set theory

The fuzzy set theory helps considerably in robust decision-making. When DM decides a decision in the crisp environment there lies many disadvantages. It does not offer more alternatives to a DM hence sometimes possess judgmental biases and vagueness. The vague or sometimes-incomplete information in crisp form may be misleading. The use of fuzzy set theory can be used in decision-making in a fuzzy environment.

The triangular fuzzy numbers (TFN) (*m*_1_, *n*_1_, *o*_1_) or trapezoidal numbers (*m*_1_, *n*_1_, *o*_1,_ *p*_1_) can be used in pairwise decision-making [88].

**Fig 2:**
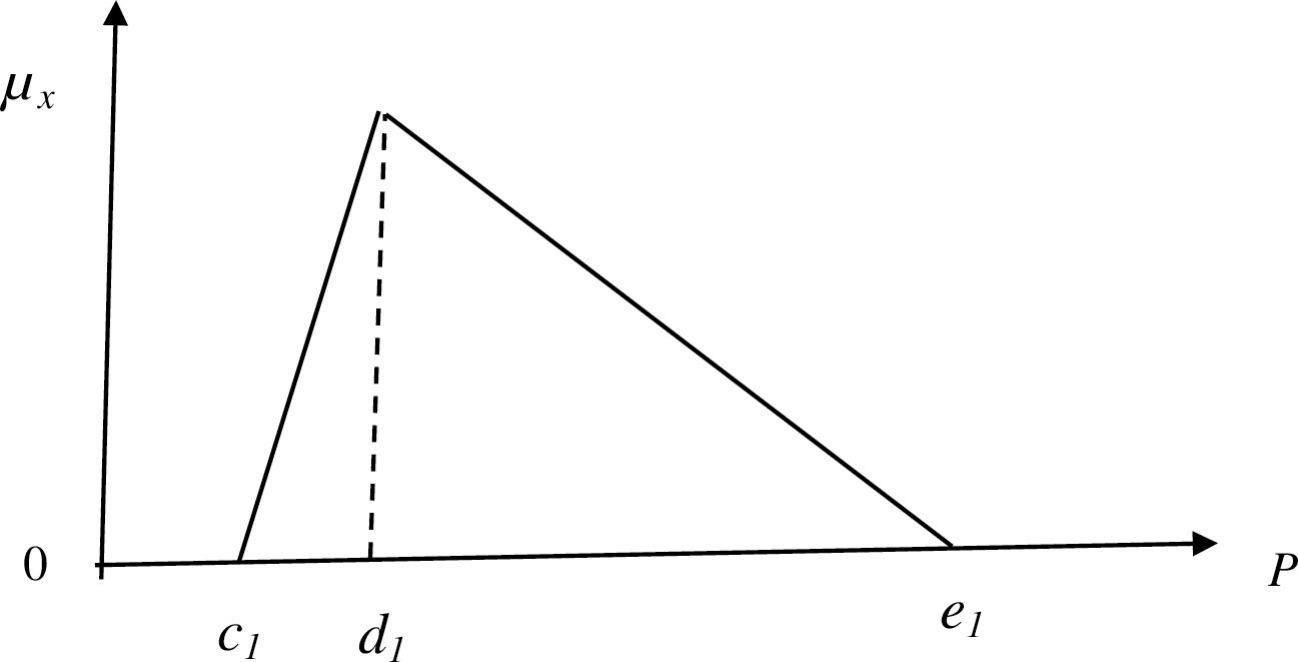
Triangular fuzzy number (*P*).

The fuzzy set theory makes use of TFNs for different arithmetic operations [89]. TFNs are represented by *P*_1_ and *P*_2_ as (*c*_1_,*d*_1_,*e*_1_) and (*c*_2_,*d*_2_,*e*_2_) respectively as shown in Fig 2. TFN can be employed to represent the fuzzy terms in gathering information, further, it can be employed in representing the uncertainty in information, vagueness. In any arithmetic operation, two TFNs are used. The arithmetic operations like subtraction, addition, division, and multiplication may be carried out using two TFNs. Such arithmetic operations can be represented by the following Eqs (4–10):

$${\tilde{P}}_{1}⊕{\tilde{P}}_{2}=(c_{1}+c_{2},\;d_{1}+d_{2},\;e_{1}+e_{2})$$ (6)

$${\tilde{P}}_{1}⊖P_{2}=(c_{1}-c_{2},d_{1}-d_{2},\;e_{1}-e_{2})$$ (7)

$${\tilde{P}}_{1}⊗{\tilde{P}}_{2}=(c_{1}c_{2},\;d_{1}d_{2},\;e_{1}e_{2})$$ (8)

$$\lambda ⊗{\tilde{P}}_{1}=\left(\lambda_{1}c_{1},\lambda_{1}d_{1},\lambda_{1}e_{1}\right)\;\mathrm{where}\;\lambda >0,\lambda \epsilon R$$ (9)

$${\tilde{P}}_{1}^{-1}=\left(\frac{1}{e_{1}},\frac{1}{d_{1}},\frac{1}{c_{1}}\right)$$ (10)

#### 4.2.2. Application of extent analysis principle in MCDM under fuzzy environment

The comparison of two TFNs, is carried out using the extent analysis principles [90]. Two sets i.e. set of objective and set for goal are considered as *Y* = {*y*_1_, *y*_2_,………,*y*_*n*_} and *Z* = {*z*_1_, *z*_2_,………,*z*_3_} respectively. Thus, using the extension principle each object is derived and extent analysis for each goal can be performed. As a result, *m* extent analysis values for each object can be derived as:

$$P_{gi}^{1},P_{gi}^{2}\ldots P_{gi}^{m},i=1,2,\ldots ,n$$ (11)

Where $P_{gi}^{j}\;(j=1,2,\ldots m)$ are TFNs and represented as (*c*,*d*,*e*). The procedure based on extent analysis [91, 92] is explained below:

***Step 1*:** *Establishing a hierarchy structure for the given goal*

The E-Learning system can be grouped into multiple levels comprising of dimensions and CSFs. The hierarchy is verified suing feedback from the DMs. It is important to frame the hierarchical structure for ranking. The final hierarchy may have a ranking of dimensions and CSFs of E-Learning at the top, followed by dimensions and CSFs of E-Learning.

***Step 2*:** *Establishing the pairwise comparison for dimension and CSFs of E-Learning using TFNs*

The dimension and CSFs of E-Learning are analyzed and compared using the feedback from DMs. The pairwise comparison of dimension and CSFs of E-Learning is finally established. In the entire pairwise comparison matrix, the TFNs are used in fixing the relationship among such pairwise comparison.

***Step 3*:** *Obtaining the value of fuzzy synthetic extent*

$$F_{i}=\sum_{j=1}^{m}P_{gi}^{j}⊗{\left[\sum_{i=1}^{n}\sum_{j=1}^{m}P_{gi}^{j}\right]}^{-1}$$ (12)

Using fuzzy summation of TFNs, *m* extent analysis values $\sum_{j=1}^{m}P_{gi}^{j}$, are obtained as:

$$\sum_{j=1}^{m}P_{gi}^{j}=(\sum_{j=1}^{m}c_{j},\sum_{j=1}^{m}d_{j},\sum_{j=1}^{m}e_{j})$$ (13)

and ${\left[\sum_{j=1}^{n}\sum_{j=1}^{m}P_{gi}^{j}\right]}^{-1}$, gives the fuzzy summation of

$P_{gi}^{j}(j=1,2,\ldots ,m)$ values are calculated as

$$\sum_{i=1}^{n}\sum_{j=1}^{m}N_{gi}^{j}=(\sum_{j=1}^{m}c_{j},\sum_{j=1}^{m}d_{j},\sum_{j=1}^{m}e_{j})$$ (14)

The inverse of the vector is obtained as:

$${\left\lceil \sum_{i=1}^{n}\sum_{j=1}^{m}P_{gi}^{j}\right\rceil }^{-1}=\left(\frac{1}{\sum_{i=1}^{n}e_{i}},\frac{1}{\sum_{i=1}^{n}d_{i}},\frac{1}{\sum_{i=1}^{n}{mc}_{i}}\right)$$ (15)

***Step 4*:** *Obtaining the degree of possibility of supremacy for two TFNs i*.*e*. *P*_2_ = (*c*_2_,*d*_2_,*e*_2_)≥*P*_1_ = (*c*_1_,*d*_1_,*e*_1_)

$$V\left(P_{2}\geq P_{1}\right)=sup\left[min(\mu_{P_{1}}\left(x\right),\mu_{P_{2}}\left(y)\right)\right],\;y\geq x$$ (16)

and can be represented as:

$$V(P_{2}\geq P_{1})=hgt\;(P_{1}\cap P_{2})=\mu_{P_{2}}(f)$$ (17)

$$\mu_{P_{2}}(f)=\left\{ \begin{array}{cc} 0 & if\;d_{2}\geq d_{1}\\ 1 & if\;c_{1}\geq e_{2}\\ \frac{c_{1}-e_{2}}{{(d}_{2}-o_{2})-(d_{1}-c_{1})} & otherwise \end{array}\right.$$ (18)

Various DMs are involved in the group decision-making for instance *K* DMs may be participating, thus the subsequent pairwise comparisons yield *n* elements. A set of *K* matrices, ${\overset{ˇ}{A}}_{k}=\{{\overset{ˇ}{p}}_{ijk}\}$, where ${\overset{ˇ}{A}}_{k}={\overset{ˇ}{p}}_{ijk}=(c_{ijk},d_{ijk},e_{ijk})$ represents the relative importance of element *i* to *j*, as derived by DM *k*. The aggregation is obtained using the Eq (19).

$$c_{ij}=min(c_{ijk}),k=1,2,\ldots k$$

$$d_{ij}=\sqrt[{k}]{\prod_{k=1}^{K}d_{ijk}}$$

$$e_{ij}=max(e_{ijk}),k=1,2,\ldots k$$ (19)

**Fig 3:**
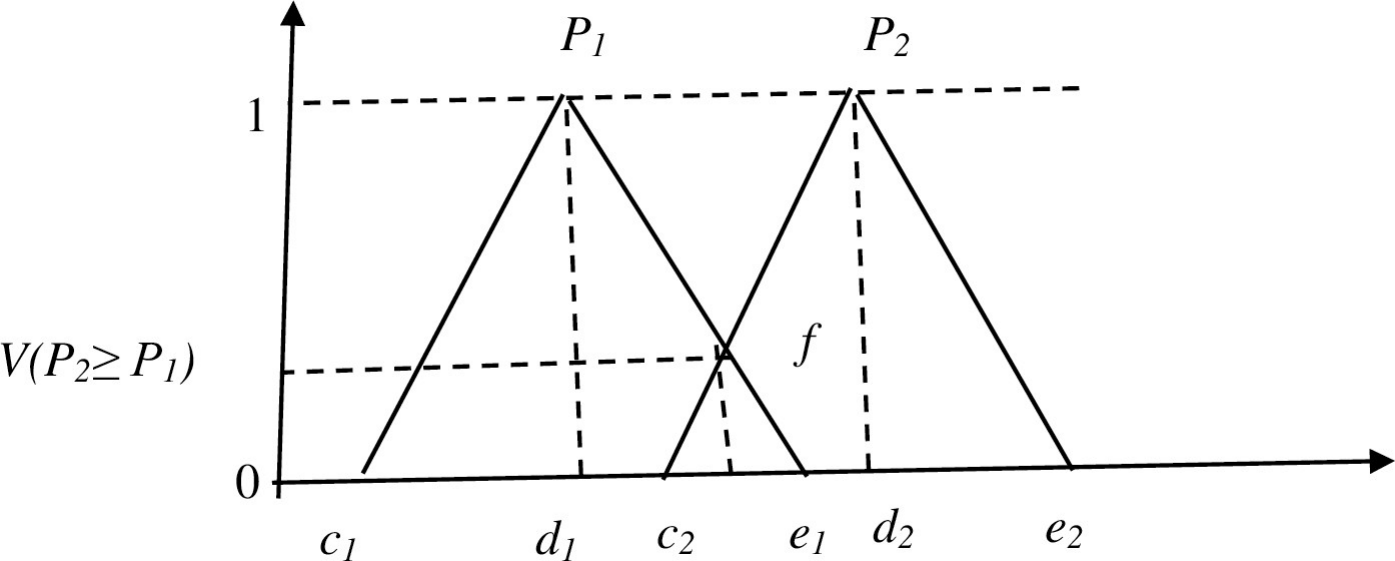
The intersection of TFNs [41].

The two TFNs i.e. (*c*_1_, *d*_1_, *e*_1_) and (*c*_2_, *d*_2_, *e*_2_) intersect at *d* which as shown in Fig 3. It also gives ordinate *d*, from the possible highest intersection between two fuzzy numbers μ*P*_1_ and μ*P*_2_ denoted as *Q*. Thus *P*_1_ and *P*_2_, are calculated through the values of *V* (*P*_1_ ≥ *P*_2_) and *V* (*P*_2_ ≥ *P*_1_).

***Step 5*:** *Obtain the degree of possibility for a given convex fuzzy number such that it is greater than k convex*

Fuzzy number *P*_1_(*i* = 1,2,….,*k*) is derived as

$$V(P\geq P_{1},P_{2}\ldots .P_{k})=V[\left(P\geq P_{1})\;and\;(P\geq P_{2}\;and\;\ldots \ldots \ldots \;and\;(P\geq P_{k})\right)]$$ (20)

$$=\min\;V\;(P\geq P_{i}),\;i=1,2,\ldots \ldots \ldots ,k$$

Considering,

$$d^{\prime }(B_{i})=\min\;V(S_{i}\geq S_{k})\;for\;k=1,2,\ldots ..,m;\;k\neq i$$ (21)

Weight vector is derived as *G*′ = (*d*′(*B*_1_),*d*′(*B*_2_),………,*d*′(*B*_*n*_))^*T*^

Such that *B*_*i*_(*i* = 1,2,…..,*n*) has *n* elements

***Step 6*:** *Obtain the normalized weight vectors*.

The normalized weight vector is calculated using Eq (22)

$$C={\left(d(B_{1}),d(B_{2}),\ldots \ldots \ldots ,d(B_{n})\right)}^{T}$$ (22)

Where *C* denotes crisp number.

***Step 7*:** *Obtaining the overall score of each CSFs dimension and its factors for the prioritization*

The overall priority weights of each dimension and CSFs of E-Learning is obtained by multiplying local weight and global weight. The global weights of dimensions and CSFs of E-Learning are arranged in descending order. The overall rank so obtained gives the required prioritization.

## 5. AHP-GDM and FAHP methodologies for prioritizing the dimensions and CSFs of E-Learning

AHP-GDM and FAHP are MCDM methodology for a pairwise comparison in the crisp environment and fuzzy environment respectively. To evaluate and prioritize the dimensions and CSFs of E-Learning, a combinatorial approach of AHP-GDM and FAHP are used. The AHP-GDM methodology offers accuracy benefits in calculating the weights of dimension and CSFs of E-Learning.

The FAHP helps to eliminate vagueness in the process of decision-making. To carry out AHP-GDM and FAHP, five DMs have been identified who have 6 years or more experience of direct involvement in training through E-Learning. Out of these five DMs, Two DMs also have E-Learning organizational experience with hardware and E-Learning software administration. The other three DMs are having core teaching experience in the E-Learning system. The DMs were persuaded to cooperate without reservation for the cause of educational research and they accepted. After explaining the AHP-GDM method, the questionnaire was given to the expert DMs physically and data was collected from them. Fig 1 describes the framework obtained through a detailed review of the literature for evaluating and prioritizing the E-Learning factors. Five DMs i.e., DM1, DM2, DM3, DM4, and DM5 assessed five dimensions that are given in Tables 3, 4, 5, 6 and 7. The following steps were followed to get the final prioritized list of dimensions and factors.

1. Defining the goal of the problem.
2. Forming hierarchical Structure of the dimensions and its factors.
3. Pairwise comparison matrices construction for each DM.
4. Synthesizing of a pairwise comparison of each dimension and its factors.
5. Check the consistency of the pairwise comparisons
6. Aggregation of DMs’ judgment.
7. Ranking of all dimensions and factors based on global weight.

**Table 3:** A pairwise comparison of E-Learning dimensions by DM1 using AHP-GDM.

Dimensions of E-Learning	SD	ID	DCD	STD	IMD	Weight
SD	1	3	2	2	1/2	0.2620
ID	1/3	1	3	1/2	1/2	0.1404
DCD	1/2	1/3	1	1/3	1/2	0.0912
STD	1/2	2	3	1	1/2	0.1926
IMD	2	2	2	2	1	0.3138

(λ_Max_ = 5.3812, RI = 1.1200, CI = 0.0953 and CR = 0.0851)

**Table 4:** A pairwise comparison of E-Learning dimensions by DM2 using AHP-GDM.

Dimensions of E-Learning	SD	ID	DCD	STD	IMD	Weight
SD	1	3	2	2	1/2	0.2620
ID	1/3	1	3	1/2	1/2	0.1404
DCD	1/2	1/3	1	1/3	1/2	0.0912
STD	1/2	2	3	1	1/2	0.1926
IMD	2	2	2	2	1	0.3138

(λ_Max_ = 5.3777, RI = 1.1200, CI = 0.0944 and CR = 0.0843)

**Table 5:** A pairwise comparison of E-Learning dimensions by DM3 using AHP-GDM.

Dimensions of E-Learning	SD	ID	DCD	STD	IMD	Weight
SD	1	3	2	2	1/2	0.2605
ID	1/3	1	2	1/3	1/2	0.1202
DCD	1/2	1/2	1	1/2	1/2	0.1038
STD	1/2	3	2	1	1/2	0.1986
IMD	2	2	2	2	1	0.3167

(λ_Max_ = 5.3197, RI = 1.1200, CI = 0.0799 and CR = 0.0714)

**Table 6:** A pairwise comparison of E-Learning dimensions by DM4 using AHP-GDM.

Dimensions of E-Learning	SD	ID	DCD	STD	IMD	Weight
SD	1	4	2	3	1/2	0.2883
ID	1/4	1	2	1/3	1/2	0.1163
DCD	1/2	1/2	1	1/2	1/3	0.0922
STD	1/3	3	2	1	1/2	0.1780
IMD	2	2	3	2	1	0.3252

(λ_Max_ = 5.4003, RI = 1.1200, CI = 0.1001 and CR = 0.0894)

**Table 7:** A pairwise comparison of E-Learning dimensions by DM5 using AHP-GDM.

Dimensions of E-Learning	SD	ID	DCD	STD	IMD	Weight
SD	1	3	3	2	1/3	0.2494
ID	1/3	1	2	1/3	1/3	0.1084
DCD	1/3	1/2	1	1/2	1/2	0.0971
STD	1/2	3	2	1	1/2	0.1883
IMD	3	3	2	2	1	0.3568

(λ_Max_ = 5.3782, RI = 1.1200, CI = 0.0945 and CR = 0.0844)

The output of each step is given below:

***Step 1*:** *Goal*

The initial step of prioritization of dimension and CSFs of E-Learning are considered as the goal of the problem.

***Step 2*:** *Hierarchical Structure*

**Fig 4:**
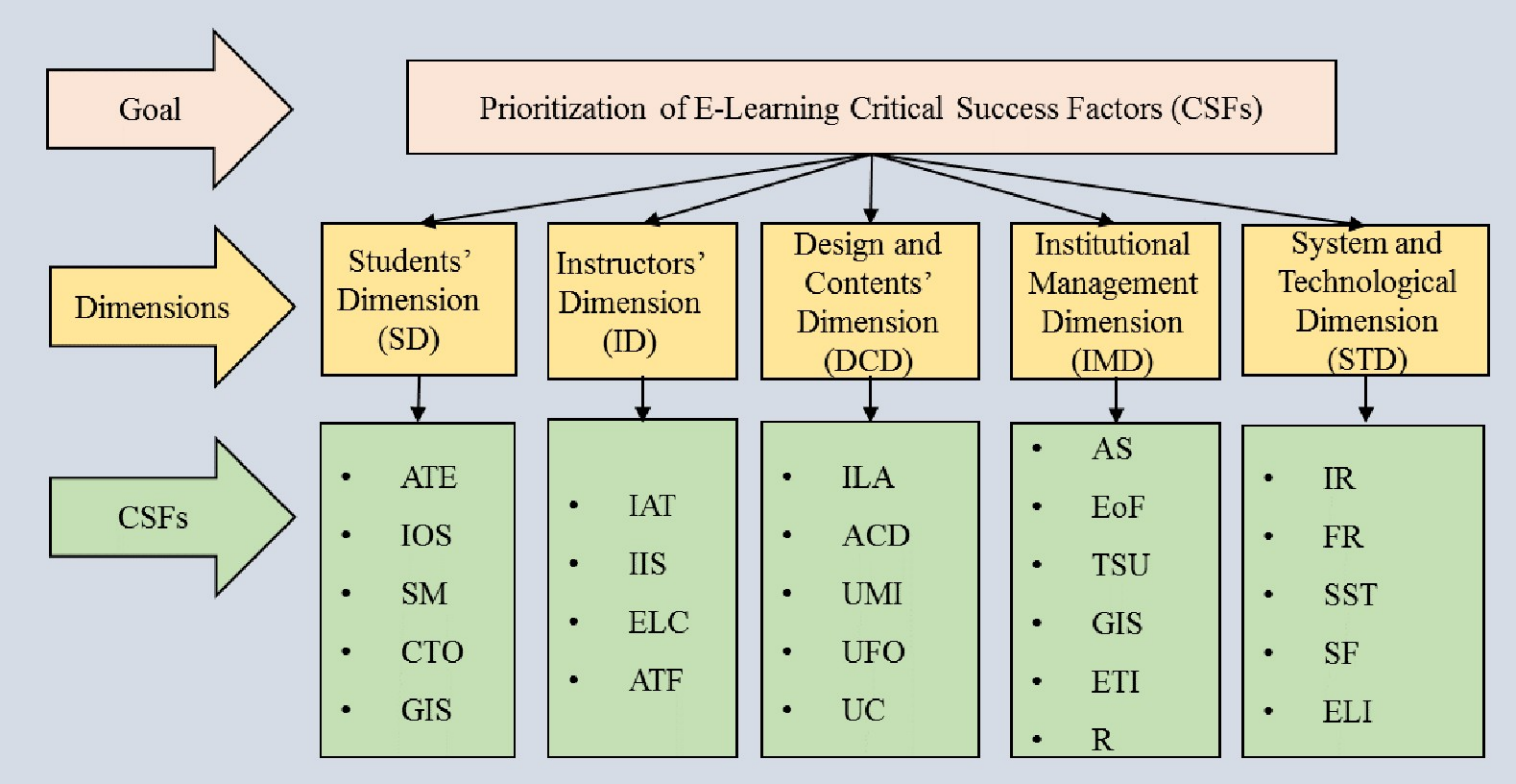
Hierarchical structure for dimensions and CSFs of E-Learning system.

The second step is dedicated to forming the hierarchical structure by establishing the dimensions and relevant factors for each dimension. Fig 4 illustrates the typical hierarchical structure for dimensions and CSFs of the E-Learning system which is developed by arranging the obtained dimensions and CSFs.

The level I indicates the prioritizing the CSFs of E-Learning. The Five dimensions identified such as Students’ Dimension (SD), Instructors’ Dimension (ID), Design and Content’s Dimension (DCD), System & Technological Dimension (STD), Institutional Management Dimension (IMD) are placed at level II, The various CSFs under different category of dimensions like Students’ Dimension (SD) consist Attitude towards E-Learning (ATE), Students’ Motivation (SM), Interaction with other Students (IOS), Commitment towards Online Studies(CTO), General Internet self-efficacy (GIS). The Instructors’ Dimension (ID) has CSFs of Instructors’ Attitude towards (IAT), Instructors’ ICT skills (IIS), Easy Language Communication (ELC), Appropriate Timely Feedback (ATF). The CSF of Design and Content’s Dimension (DCD) are found to be Appropriate Course Design (ACD), Interactive Learning Activity (ILA), Use of Multimedia Instruction (UMI), User–Friendly Organized (UFO), Understandable Content (UC). The System & Technological Dimension (STD) consist of CSFs like Appropriate System (AS), Ease of Access (EoA), Good Internet Speed (GIS), Technical Support for Users (TSU), Reliability (R), Efficient Technology Infrastructure (ETI), Finally, the Institutional Management Dimension (IMD) possess the CSFs of Infrastructure Readiness (IR), Financial Readiness (FR), Training for User (SST), Support for Faculty (SF), Ethical & Legal Issues (ELI).

***Step 3*:** *Pairwise Comparison Matrices*

After constructing the hierarchical structure, the relative contribution of each CSFs and their dimensions are obtained by making a pairwise comparison of each DM. In AHP-GDM, an expert’s opinion is used for getting final opinions. The feedback from the DMs’ plays an important role in getting the pairwise comparison. Generally in AHP, a single DM is considered sufficient to provide a decision. However, single DM may some-time gives an ambiguous judgment. To remove this error in decision-making, group decision-making (GDM) has been employed. Five DMs were participated to give their expert opinion in framing a pairwise comparison, which helped in getting a decision without bias. The scale used for a pairwise comparison matrix is depicted in Table 2 [86]. The pairwise comparison matrix Tables 3–7 are further synthesized using the geometric mean method to give the final pairwise comparison matrix. The matrix so obtained using GDM has more accuracy compared to a single DM.

***Step 4*:** *Synthesizing of Pairwise Comparison*

After completing a pairwise comparison of various dimensions of E-Learning, the results are tabulated as an output matrix (Tables 3–7). It refers to the relative contribution of one element over the other. Since the group decision-making plays an important role in more accurate decision-making. Hence, the pairwise comparison matrix using GDM is synthesized using the geometric mean. The geometric mean method is preferred over the arithmetic mean method due to its non-reciprocity of the pairwise matrix. The pairwise comparison matrix derived by each DM is synthesized and presented in Table 8.

**Table 8:** Synthesized pairwise comparison of E-Learning dimensions by DM1 to DM5 using GM.

Dimensions of E-Learning	SD	ID	DCD	STD	IMD	Weight
SD	1.00	3.18	2.17	2.17	0.46	0.2603
ID	0.31	1.00	2.35	0.39	0.43	0.1251
DCD	0.46	0.43	1.00	0.43	0.46	0.0964
STD	0.46	2.55	2.35	1.00	0.50	0.1900
IMD	2.17	2.35	2.17	2.00	1.00	0.3282

(λ_Max_ = 5.3315, RI = 1.1200, CI = 0.0829 and CR = 0.0740)

***Step 5*:** *Check Consistency*

Consistency level of the obtained values after a pairwise comparisons are checked with the help of Consistency level (CI) and Random Index (RI) using Eq 3.

***Step 6*:** *Aggregation of Judgement*

The next step was to aggregate the multiple values provided by different DMs in the relevant matrix into a single value. Thus, the weighted GM method was applied to the aggregate judgment of all five DMs. After the single value was obtained the same process was repeated to find the synthesize value of each dimension and factors after aggregation.

***Step 7*:** *Ranking*

In the final step, the factors from all dimensions were ranked based on their global weights, which was determined as a relative contribution to the E-Learning success. The AHP pairwise matrix offers local weights for each element. The product of such local weight of E-Learning dimension and E-Learning CSFs are obtained using the following relations:

Global weights = ∑(Local weight for dimension *i* x local weight for factor *j* with respect to dimension *i*)

The global weights so obtained are arranged in descending order to prioritize the CSFs of E-Learning that contributes the maximum influence in the E-Learning success. Thus it has been seen that AHP-GDM will help to get an overall ranking of factors [91].

Table 9 shows the composite weights of each E-Learning CSFs attained through AHP-GDM.

**Table 9:** Composite rank and weight of dimension and CSFs of E-Learning using AHP-GDM.

Dimensions of E-Learning	Dimension weight	CSFs of E-Learning	Local Weights	Global Weights	Rank
SF	0.2603	ATE	0.3493	0.0901	2
		SM	0.1628	0.0408	8
		IOS	0.1202	0.0336	11
		CTO	0.2828	0.0739	5
		GIS	0.0826	0.0219	16
IF	0.1251	IAT	0.3057	0.0386	9
		IIS	0.1036	0.0131	19
		ELC	0.1829	0.0234	15
		ATF	0.4039	0.0500	6
DCF	0.0964	ILA	0.1019	0.0101	22
		ACD	0.3940	0.0386	10
		UMI	0.1362	0.0136	18
		UFO	0.2759	0.0264	13
		UC	0.0763	0.0076	24
STF	0.1900	AS	0.2615	0.0506	7
		EoA	0.0391	0.0080	23
		TSU	0.1245	0.0251	14
		GIS	0.0608	0.0123	20
		ETI	0.4750	0.0872	4
		R	0.0337	0.0068	25
IMF	0.3282	IR	0.2758	0.0910	3
		FR	0.5444	0.1730	1
		SST	0.0529	0.0193	17
		SF	0.0948	0.0336	12
		ELI	0.0317	0.0112	21

In the same way, FAHP is employed to establish the weighs of dimension and CSFs to get the ranking. The TFN based scale as shown in Table 10 has been used in getting the weights of factors and their dimensions. The methodology explained in section 3 is followed to found the local weights and global weight. Table 11 demonstrates the weights of dimensions. Table 12 shows the weight of each factor. The prioritization obtained using MCDM is further compared and shown in Table 13.

**Table 10:** TFN scale for a pairwise comparison using FAHP [87].

Linguistics Scale for Importance	TFN Scale	TFN Reciprocal Scale
Equally Importance	(1/2,1,3/2)	(2/3,1,2)
Weakly more importance	(1,3/2,2)	(1/2,2/3,1)
Strongly more importance	(3/2,2,5/2)	(2/5,1/2,2/3)
Very strongly more importance	(2,5/2,3)	(1/3,2/5,1/2)
Absolutely more importance	(5/2,3,7/2)	(2/7,1/3.2/5)

**Table 11:** A pairwise comparison of E-Learning dimensions using FAHP.

Dimensions of E-Learning	SD	ID	DCD	STD	IMD	WEIGHT
SD	(1,1,1)	(2/3,1,1)	(2/3,1,3/2)	(2/3, 2,3/2)	(1,1,1)	0.2392
ID	(1,1,3/2)	(1,1,1)	(1,1,1)	(2/3, 1,3/2)	(2/3, 2/3,3/2)	0.1791
DCD	(2/3,1,3/2)	(1,1,1)	(1,1,1)	(2/3, 2,3/2)	(1,1,1)	0.1844
STD	(2/5,1/2,2/3)	(2/3,1,3/2)	(2/3,1,3/2)	(1,1,1)	(2/3, 2/3,3/2)	0.1579
IMD	(1,1,1)	(2/3,3/2,3/2)	(1,1,1)	(2/3,3/2,3/2)	(1,1,1)	0.2393

**Table 12:** Composite rank and weight of dimension and CSFs of E-Learning using FAHP.

Dimensions of E-Learning	Dimension Weightage	CSFs of E-Learning	Criteria Weights		Rank
			Local Weights	Global Weights	
SD	0.2392	ATE	0.3227	**0.077**	**2**
		SM	0.2197	**0.053**	**6**
		IOS	0.1506	**0.036**	**12**
		CTO	0.1725	**0.041**	**11**
		GIS	0.1344	**0.032**	**15**
ID	0.1791	IAT	0.2757	**0.049**	**7**
		IIS	0.1485	**0.027**	**19**
		ELC	0.2651	**0.047**	**8**
		ATF	0.3107	**0.056**	**5**
DCD	0.1844	ILA	0.1747	**0.032**	**14**
		ACD	0.3415	**0.063**	**4**
		UMI	0.1616	**0.030**	**16**
		UFO	0.2338	**0.043**	**10**
		UC	0.0886	**0.016**	**24**
STD	0.1579	AS	0.2210	**0.035**	**13**
		EoA	0.1596	**0.025**	**20**
		TSU	0.1535	**0.024**	**22**
		GIS	0.1556	**0.025**	**21**
		ETI	0.1749	**0.028**	**18**
		R	0.1354	**0.021**	**23**
IMD	0.2393	IR	0.2863	**0.069**	**3**
		FR	0.3333	**0.080**	**1**
		SST	0.0637	**0.015**	**25**
		SF	0.1939	**0.046**	**9**
		ELI	0.1229	**0.029**	**17**

**Table 13:** A comparison of composite weights and rank of dimension and CSFs of E-Learning using AHP-GDM and FAHP.

Dimensions of E-Learning	Dimension Weightages		CSFs	Local Weights		Global Weights		Overall Ranking	
	AHP-GDM	FAHP		AHP-GDM	FAHP	AHP	FAHP	AHP	FAHP
SD	0.2603	0.2392	ATE	0.3493	0.3227	0.0901	0.077	2	2
			SM	0.1628	0.2197	0.0408	0.053	8	6
			IOS	0.1202	0.1506	0.0336	0.036	11	12
			CTO	0.2828	0.1725	0.0739	0.041	5	11
			GIS	0.0826	0.1344	0.0219	0.032	16	15
ID	0.1251	0.1791	IAT	0.3057	0.2757	0.0386	0.049	9	7
			ILS	0.1036	0.1485	0.0131	0.027	19	19
			ELC	0.1829	0.2651	0.0234	0.047	15	8
			ATF	0.4039	0.3107	0.0500	0.056	6	5
DCD	0.0964	0.1844	ILA	0.1019	0.1747	0.0101	0.032	22	14
			ACD	0.3940	0.3415	0.0386	0.063	10	4
			UMI	0.1362	0.1616	0.0136	0.030	18	16
			UFO	0.2759	0.2338	0.0264	0.043	13	10
			UC	0.0763	0.0886	0.0076	0.016	24	24
STD	0.1900	0.1579	AS	0.2615	0.2210	0.0506	0.035	7	13
			EOA	0.0391	0.1596	0.0080	0.025	23	20
			TSU	0.1245	0.1535	0.0251	0.024	14	22
			GIS	0.0608	0.1556	0.0123	0.025	20	21
			ETT	0.4750	0.1749	0.0872	0.028	4	18
			R	0.0337	0.1354	0.0068	0.021	25	23
IMD	0.3282	0.2393	IR	0.2758	0.2863	0.0910	0.069	3	3
			FR	0.5444	0.3333	0.1730	0.080	1	1
			SST	0.0529	0.0637	0.0193	0.015	17	25
			SF	0.0948	0.1939	0.0336	0.046	12	9
			ELI	0.0317	0.1229	0.0112	0.029	21	17

## 6. Results and discussion

The present paper help to establish the influence of dimension and CSFs of E-Learning using MCDM. Both AHP and FAHP methods are capable of delivering the fruitful results. In case of AHP, the results may further be improved using GDM is preferred over individual decision-making. The prioritized CSFs may prove to be helpful to the administrators to derive, monitor and control the E-Learning system successfully. Generally, the E-Learning infrastructure involves huge investment for providing the latest infrastructure which includes software and hardware. To make it successful these CSFs are essentially being controlled. The results obtained by both methods i.e. AHP-GDM and FAHP are compared to get accurate ranking. E-Learning stakeholders such as the institute of higher education may find the prioritization of CSFs useful AHP-GDM and FAHP's combinatorial approach can be used to rank and extract weights. It is useful to compare the weights of all factors with AHP-GDM and FAHP to understand the importance of each CSF.

The found results show the priority of twenty-five factors. The global weights attained are organized in a decreasing manner. The global weights of top five factors thus obtained are: 0.1750 **>** 0.0919 > 0.0906 > 0.0886 > 0.0733. Where ‘>‘ indicates more preference over the criteria. The CSFs of E-Learning contributing significantly to the success of E-Learning are 1. Financial Readiness (FR) 2. Efficient Technology Infrastructure (ETI) 3. Attitude towards E-Learning (ATE) 4. Infrastructure Readiness (IR) and 5. Reliability (R).

However, based on the FAHP the weights and its group classification are changing. The obtained results shows priority of first five CSFs as: 0.08 > 0.077 >0.069 > 0.063 > 0.056. Where ‘>‘ indicates more preference over criteria. The CSFs of E-Learning with reference to its comparative contribution to the success of E-Learning is found as 1. Financial Readiness (FR) 2. Attitude towards E-Learning (ATE) 3. Infrastructure Readiness (IR) 4. Appropriate Course Design (ACD) 5. Appropriate Timely Feedback (ATF).

**Fig 5:**
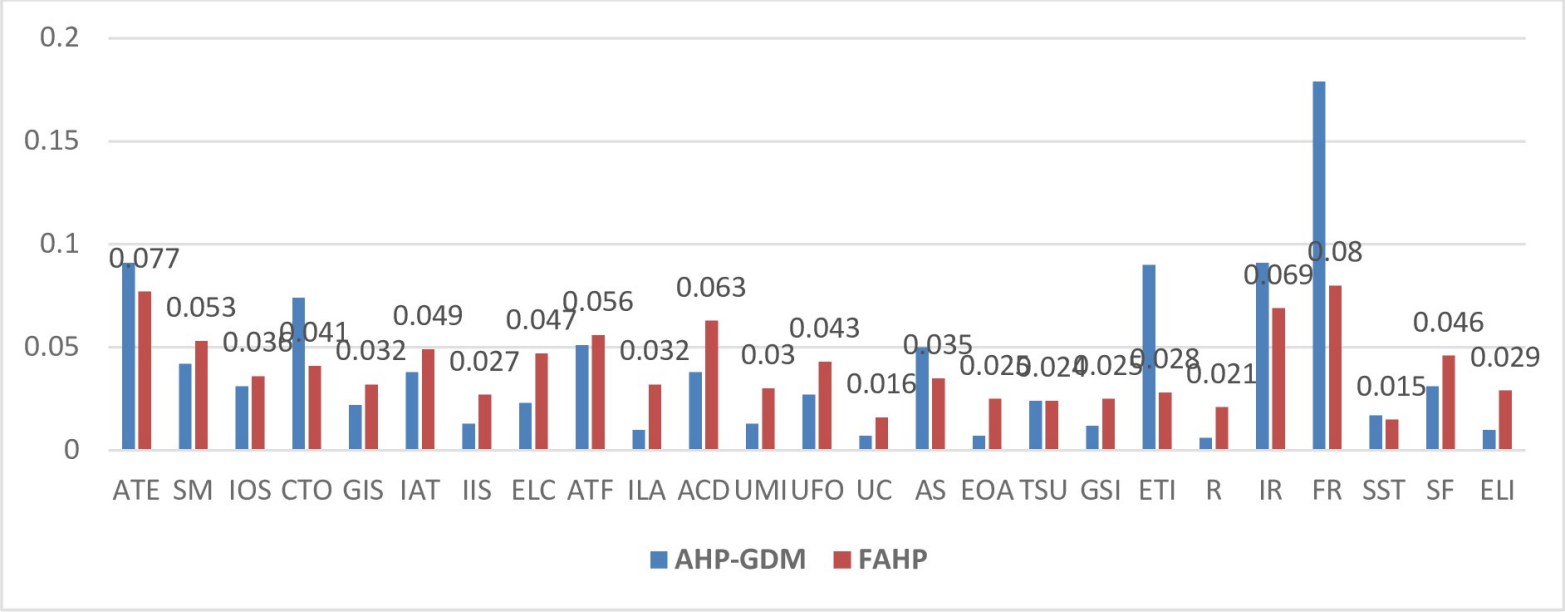
A comparison of weights of CSFs of E-Learning using AHP-GDM and FAHP.

**Fig 6:**
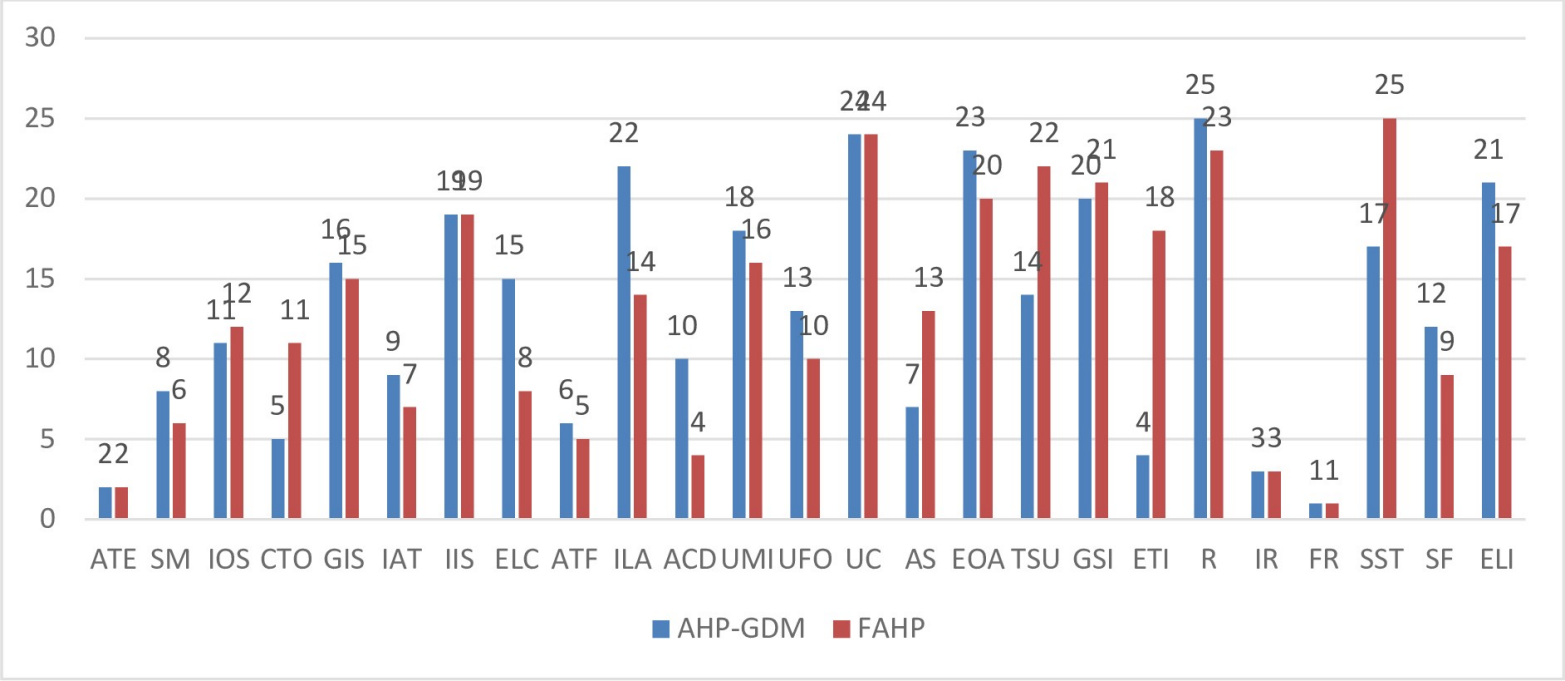
A comparison of ranks CSFs of E-Learning using AHP-GDM and FAHP.

Fig 5 demonstrates the comparison of CSFs weights of E-Learning obtained with MCDM. Similarly, it is also possible to compare rank E-Learning factors using MCDM. Fig 6 displays the comparison of E-Learning rankings obtained from MCDM.

Based on the weights obtained by MCDM two prioritization sequences are obtained. The various weights of five E-Learning dimensions obtained by MCDM are as: 0.3283 > 0.2603 > 0.1900 > 0.1251 > 0.0964 where ‘>‘ represents ‘more importance for IMD > SD > STD > ID > DCD i.e. Institutional Management Dimension (IMD), Students’ Dimension (SD), System and Technological Dimension (STD), Instructors' Dimension (ID), and Design and Contents’ Dimension (DCD), Similarly, The various weights of five E-Learning dimensions obtained by FAHP methodology are as 0.2393 > 0.2392 > 0.1844 > 0.1791 > 0.1579 > where ‘>‘ represents ‘more importance’ for IMD > SD > DCD > ID > STD i.e. for Institutional Management Dimension (IMD), Students’ Dimension (SD), Design and Contents’ Dimension (DCD), Instructors' Dimension (ID), and System and Technological Dimension (STD).

Similarly, the prioritized rank of CSFs by MCDM is obtained. To systemize the prioritizations obtained by both methodologies, it is further grouped into three categories i.e. high influence, moderate influence, and low influence. Based on the DMs’ expert opinion prioritized CSFs of E-Learning are using AHP-GDM are classified into three groups as shown in Table 14. The Financial Readiness (FR), Efficient Technology Infrastructure (ETI), Attitude towards E-Learning (ATE), Infrastructure Readiness (IR) and Commitment towards Online Studies (CTO) classified as group I having high influence, The Appropriate Timely Feedback (ATF), Appropriate System (AS), Students’ Motivation (SM), Instructors’ Attitude towards E-Learning (IAT), Appropriate Course Design (ACD), Interaction with other Students (IOS), Support for Faculty (SF) are classified as Gr.II having moderate influence. Whereas, the User-Friendly Organized (UFO), Easy Language Communication (ELC), Technical Support for Users (TSU), General Internet self-efficacy (GIS), Training for User (SST), Instructors’ ICT Skills (IIS), Use of Multimedia Instruction (UMI), Good Internet Speed (GIS), Interactive Learning Activity (ILA), Ethical & Legal Issues (ELI), Ease of Access (EoA), Understandable Content (UC), Reliability (R) are classified as low influences group. Similarly, CSFs of E-Learning using FAHP are also grouped which is shown in Table 14.

**Table 14:** Classification of CSFs of E-Learning into varying degree of influence-group.

CSFs of E-Learning	Classification of CSFs in Influence Group	
	AHP-GDM Group	FAHP Group
FR	I	I
ETI	I	I
ATE	I	I
IR	I	I
CTO	I	I
ATF	I	I
AS	II	II
SM	II	II
IAT	II	II
ACD	II	II
IOS	II	II
SF	II	II
UFO	III	II
ELC	III	II
TSU	III	II
GIS	III	II
SST	III	III
IIS	III	III
UMI	III	III
GIS	III	III
ILA	III	III
ELI	III	III
EoA	III	III
UC	III	III
R	III	III

## 7. Limitations and scope for future research

The assessment and prioritization of E-Learning’s dimensions and CSFs influence its success. Nevertheless, depending on the different financial, social, and regional conditions, the effect of CSFs may change when compared with the global situation. CSFs of E-Learning has been established for Saudi Arabia in the current research after evaluation and prioritization. However, the priorities obtained for 25 CSFs are limited to this country. Besides that, this present research has provided a robust AHP-GDM and FAHP based methodology for assessing and prioritizing the CSFs. Nonetheless, the results may not be generalized, as the assessment was based on DMs. By employing GDM, the biases have been removed to a great extent; however, a more accurate judgment is required. Hence, TOPSIS and Fuzzy TOPSIS may be employed to remove vagueness and biases in judgment and optimize the CSFs that influence E-Learning.

## 8. Conclusions

E-Learning and mobile learning (M-Learning) are the subset of digital learning (D-Learning). These days, students are well conversant with computer and mobile usage hence find both the version easy and comfortable for their learning. Both E-Learning and M-Learning have their users varying with age, sex, region, social culture etc. The E-Learning provides speedy access with multimedia to demonstrate much more understanding to users. The success of E-Learning is highly depending upon the CSFs. The CSFs affecting the E-Learning success are many hence it is essential to evaluate and prioritize them so that the management providing E-Learning can invest, regulate E-Learning infrastructure in an effective manner.

As it is evident that, CSFs play a key role in E-Learning success. Hence, investigations related to the impact of dimensions and CSFs on learning and teaching are mandatory. After assessing the impact of each CSF, the various stakeholders, such as university authority, students, and instructors, will be able to control the negative effects of each of these E-Learning factors and their dimensions. The MCDM approach could be fruitful in categorizing each dimension and CSF of the E-Learning system. This categorization of factors will help stakeholders to decide the strategic policy for the resource management of money and time in creating and enhancing service infrastructure to improve the teaching-learning process.

## 9. Recommendation of effective E-Learning implementation for students, instructors and developers

It is recommended to evaluate and control CSFs of E-Learning in an effective manner by utilizing resources (Hardware and Software). It is further recommended to provide proper training of the E-Learning system to students and instructors to obtain learning and teaching skills respectively. Lack of training can prevent effective utilization and possible benefits from the system. Professional training can enhance the instructors’ capabilities to access virtual class facilities. It helps in increasing multimedia in the E-Learning. The instructor will be able to manage various miscellaneous technical applications like assignments and tests, discussion board, course messages, and grading for effective course management. The well-trained students can achieve full benefits of the system. Instructors can also adopt suitable teaching practices to provide a rich learning environment by introducing teaching notes, video, and multimedia. Instructors can also use social media to enhance their alliance with students. They can allow them to share their E-Learning system experience through various social media, such as Facebook, Twitter or YouTube.

The developers in consultation with instructors must develop various learning activities to enhance alliance between instructors and students. They can also develop purposeful and effective learning activities that can motivate the stakeholders to use the system effectively. In order to enhance the interest of students, instructors and other users, audio, video and multimedia content can be imbibed in the E-Learning. The developers may develop user-friendly environment by providing an easy and handy applications like emails, course messages, frequently asked questions (FAQs).

Universities can manage to provide special budgets for E-Learning units to purchase new or upgrade technological infrastructure. The installation of enhanced high-speed internet bandwidth of the E-Learning system can provide ease in usage to the full extent. Furthermore, upgraded software, sufficient laboratories with high computer inventory with uninterrupted networking amenities will boost the motivation of students and instructors.
